# Urinary placental growth factor stability as a critical factor in the reliability of preeclampsia diagnosis

**DOI:** 10.11613/BM.2026.020701

**Published:** 2026-04-15

**Authors:** Eva Martinez-Marzo, Maria Peran, Juan Lerma-Irureta, Ana Medel-Martinez, Cristina Paules, Daniel Oros, Marta Fabre

**Affiliations:** 1 Instituto de Investigación Sanitaria de Aragón (IIS Aragón), Zaragoza, Spain; 2 Biochemistry Department, University Clinical Hospital Lozano Blesa, Zaragoza, Spain; 3 Obstetrics Department, University Clinical Hospital Lozano Blesa, Zaragoza, Spain; 4 Red RICORS “Primary Care Interventions to Prevent Maternal and Child Chronic Diseases of Perinatal and Developmental Origin”, RD21/0012/0001, Instituto de Salud Carlos III, Madrid, Spain; 5 University of Zaragoza, Zaragoza, Spain

**Keywords:** placental growth factor, preanalytical phase, preeclampsia, stability, urine

## Abstract

**Introduction:**

Placental growth factor (PlGF) is a key biomarker for diagnosing and predicting preeclampsia (PE). While serum-based PlGF assays are well established, urine has emerged as a promising non-invasive alternative matrix. However, the absence of urinary PlGF stability data remains a major preanalytical limitation. This study aimed to assess urinary PlGF stability under common preanalytical conditions, including refrigerated storage and a double freeze-thaw cycle.

**Materials and methods:**

A prospective study was conducted using urine samples from ten pregnant women. Each sample was processed under standard laboratory conditions and aliquoted into five tubes. One aliquot was immediately frozen at - 80 °C (T0), three were stored at 2-8 °C for 48, 96, and 168 hours before freezing, and one underwent a double freeze-thaw cycle. Urinary PlGF concentrations were measured using the Elecsys PlGF immunoassay on Roche Cobas e801 analyzer. Percent degradation (PD%) was calculated relative to baseline. A linear regression model was applied to estimate the time to exceed a maximum permissible instability (MPI) of ± 10%.

**Results:**

Urinary PlGF remained stable at 2-8 °C for up to 48 hours, with a mean PD% of - 6% (95% confidence interval (CI): - 9.1 to - 2.8). The regression model (PD% = - 0.0834 x Time (h)) estimated the - 10% threshold at approximately 120 hours. After a double freeze-thaw cycle, the mean PD% was - 1.5% (95% CI: - 3.4 to 0.4%).

**Conclusions:**

Urinary PlGF shows acceptable stability for up to 48 hours under refrigeration and is stable over two freeze-thaw cycles. These findings provide essential preanalytical data supporting its potential use in clinical and research settings.

## Graphical abstract

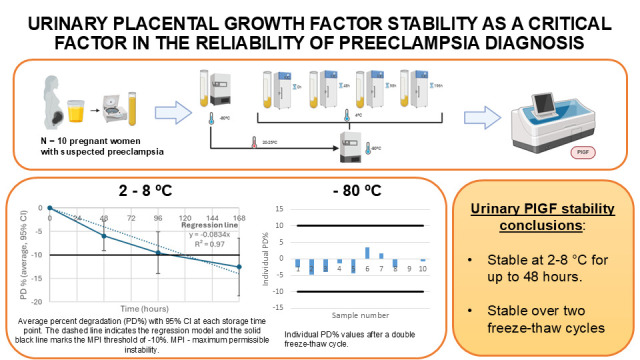

## Highlights

• Urinary placental growth factor (PlGF) may support diagnosis and prediction of preeclampsia

• First structured study assessing PlGF stability in urine samples

• The stability protocol used improves consistency across laboratories

• Urinary PlGF is stable for up to 48 hours when stored at 2-8 °C

• Urinary PlGF remains stable after two freeze-thaw cycles

## Introduction

Preeclampsia (PE) is a leading cause of maternal and perinatal morbidity and mortality worldwide (*1*). It is characterized by placental dysfunction and typically manifests after 20 weeks of gestation with new-onset hypertension and proteinuria (*2*). Early and accurate diagnosis is essential to reduce associated complications. The pathophysiology of PE has been strongly linked to an imbalance between angiogenic and antiangiogenic factors. Serum concentrations of soluble fms-like tyrosine kinase-1 (sFlt-1), an antiangiogenic molecule, are elevated in women with PE, while free circulating concentrations of placental growth factor (PlGF), a proangiogenic protein, are decreased (*3*). The serum sFlt-1/PlGF ratio has been incorporated into clinical algorithms and is recommended by international guidelines due to its high negative predictive value for PE diagnosis (*4*, *5*). Additionally, serum PlGF alone has demonstrated notable diagnostic performance (*6*, *7*).

While serum remains the standard matrix for angiogenic markers (sFlt-1 and PlGF), urine has gained increasing attention as a promising alternative sample. Its non-invasive and spontaneous collection, greater patient acceptability, and feasibility for repeated testing make it especially suitable for use in non-specialized or low-resource settings (*8*, *9*). Several studies have investigated urinary biomarkers for PE diagnostic. Proteomic analyses have identified candidates such as SERPINA1, uromodulin, albumin fragments and fibrinogen-derived peptides, which reflect endothelial and renal dysfunction in PE (*10*). Other analytes like activin A, inhibin A and sFlt-1 have also shown potential, particularly in multimarker models. However, PlGF has emerged as one of the most promising urinary biomarkers, showing consistent diagnostic performance even without creatinine adjustment (*11*).

A thorough understanding of an analyte’s properties is essential prior to its clinical application. Assessing its stability under real-world preanalytical conditions is critical to ensure reliable measurement. Clinical laboratories often depend on published literature and manufacturer specifications to define acceptable storage times (*12*). Multiple studies have evaluated the stability of PlGF in serum over periods ranging from 48 hours to 30 days; however, their findings vary depending on storage conditions, analytical methods, and stability criteria (*13*-*15*). To our knowledge, no study has systematically assessed the stability of PlGF in urine, highlighting a key preanalytical gap that must be addressed before this matrix can be reliably implemented in clinical or research settings.

The aims of this study were to assess the stability of PlGF in urine samples stored refrigerated at 2-8 °C for up to seven days as well as after a double freeze-thaw cycle, reflecting typical preanalytical conditions encountered in hospital settings.

## Materials and methods

### Subjects

This prospective study was carried out at the University Clinical Hospital Lozano Blesa (Zaragoza, Spain) within the framework of project PI22/143, supported by the Instituto de Salud Carlos III (ISCIII), Government of Spain. Following the protocol published by the Spanish Society of Laboratory Medicine (SEQC-ML) for the stability testing of biochemical analytes, ten urine samples were collected from pregnant women with suspected PE at various gestational ages (*16*). Sample collection was conducted between January and February 2025 and laboratory analysis was performed in April 2025.

The study was approved by the Research Ethics Committee of the Community of Aragón (C.I. PI19/346 and PI23/102) and all patients provided written informed consent.

### Methods

**Figure 1:**
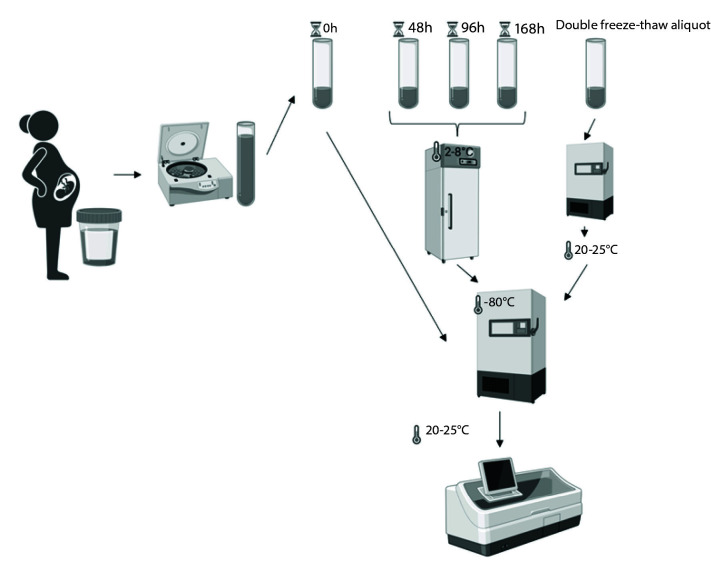
Flowchart of the stability study process from sampling to analysis. The figure was created using BioRender.com.

From each participant, 10 mL of midstream urine was collected in sterile, additive-free VACUETTE Z urine no additive 10 mL tubes (Greiner Bio-One GmbH, Kremsmünster, Austria). All urine samples were centrifuged at 3500 rpm for five minutes at room temperature and the supernatant was aliquoted into five sterile round-bottom polystyrene tubes (500 μL each) (Deltalab, Barcelona, Spain). One aliquot was immediately frozen at - 80 °C (T_0_), while the remaining three were stored refrigerated at 2-8 °C for 48, 96 and 168 hours, respectively, before being frozen at - 80 °C. An additional aliquot from each participant underwent a double freeze-thaw cycle to simulate a common preanalytical condition in research. All double freeze-thaw aliquots were frozen at - 80 °C immediately after collection (T_0_). Four days later, each aliquot was completely thawed at room temperature for approximately 30 minutes, ensuring full homogenization, and subsequently refrozen at - 80 °C, completing the first freeze–thaw cycle. The second thaw occurred on the day of laboratory analysis, when all aliquots were thawed simultaneously and analyzed in a single batch within two hours to minimize analytical variability. Because samples were collected on different dates, the interval between the second freezing and final thaw varied from 38 to 80 days. A flowchart illustrating the sample processing workflow is presented in Figure 1.

Urinary PlGF concentrations were measured using the Elecsys PlGF immunoassay (lot 796564), run on Roche Cobas e801 analyzer (Roche Diagnostics, Basel, Switzerland). As no immunoassay is currently validated for urinary PlGF measurement, analytical performance of the system was monitored using routine serum internal quality controls at low (≈ 50 pg/mL) and high (≈ 100 pg/mL) concentration levels, showing inter-assay coefficients of variations (CVs) of 3.1% and 3.3%, respectively. All samples were analyzed in duplicate, and the average of both replicates was used in all calculations.

### Statistical analysis

For each time point, the percent degradation (PD%) was calculated relative to baseline using the following formula: PD% = ((T_x_ – T_0_) / T_0_) x 100, where T_0_ is the baseline PlGF concentration and T_x_ the concentration at the specified time point. The mean PD%, standard deviation (SD) and 95% confidence interval (CI) were calculated for each storage condition. According to the SEQC-ML protocol, an analyte was considered stable if the mean PD% ± 95% CI remained within the maximum permissible instability (MPI) (*16*). An MPI threshold of ± 10% was adopted for defining analyte stability.

To model the degradation trend over time, a linear regression analysis was performed using the PD% values. The regression line was constrained to pass through the origin, in accordance with the SEQC-ML comprehensive stability protocol, reflecting the biological assumption of no degradation at time zero (*16*). The resulting regression equation was: PD% = - 0.0834 x Time (h), with a coefficient of determination R^2^ = 0.97.

All statistical analyses were performed using Jamovi v2.6 (The jamovi project, Sydney, Australia) and Microsoft Excel 365 MSO version 2505 (Microsoft Corporation, Redmond, USA).

## Results

**Figure 2:**
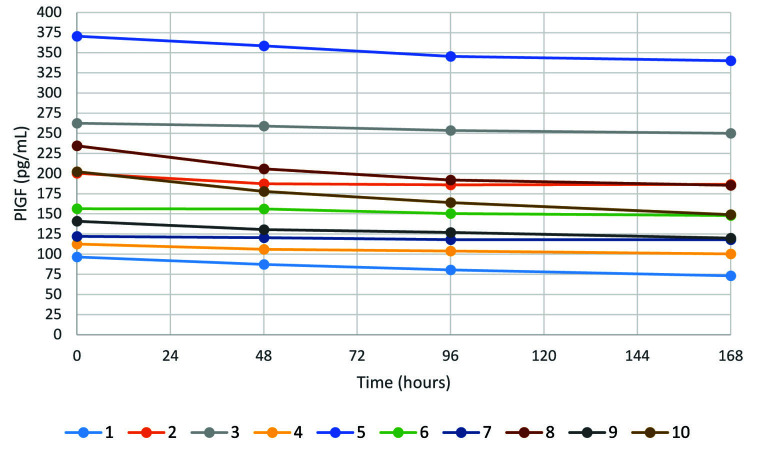
Urinary PLGF concentrations for all samples at each time point. PIGF - placental growth factor.

Urinary stability of PlGF was evaluated in samples obtained from ten pregnant women. Baseline concentrations ranged from 97 to 371 pg/mL. The mean PlGF concentration for each individual sample at every storage time point is shown in Figure 2, allowing a visual comparison of analyte behavior over time.

To assess analyte stability, the PD% relative to the baseline value was calculated for each sample after 48, 96 and 168 hours of refrigerated storage at 2-8 °C with the corresponding average PD% and 95% CI (Table 1).

**Table 1:** Individual percent degradation (PD%) values and group means with 95% confidence intervals for urinary PlGF concentrations

**Individual PD%**	**48 hours**	**96 hours**	**168 hours**
Sample 1	- 9.5%	- 16.7%	- 24.3%
Sample 2	- 6.5%	- 7.2%	- 7%
Sample 3	- 1.3%	- 3.4%	- 4.8%
Sample 4	- 5.8%	- 7.6%	- 10.8%
Sample 5	- 3.2%	- 6.8%	- 8.2%
Sample 6	- 0.3%	- 3.5%	- 5.1%
Sample 7	- 1.2%	- 3.3%	- 3.3%
Sample 8	- 12.2%	- 18.1%	- 20.9%
Sample 9	- 7.5%	- 9.9%	- 14.9%
Sample 10	- 12.1%	- 19%	- 26.4%
Average PD% (95% CI)	- 6% (- 9.1 to - 2.8)	- 9.6% (- 14 to - 5.1)	- 12.6% (- 18.7 to - 6.4)
PIGF - placental growth factor.			

**Figure 3:**
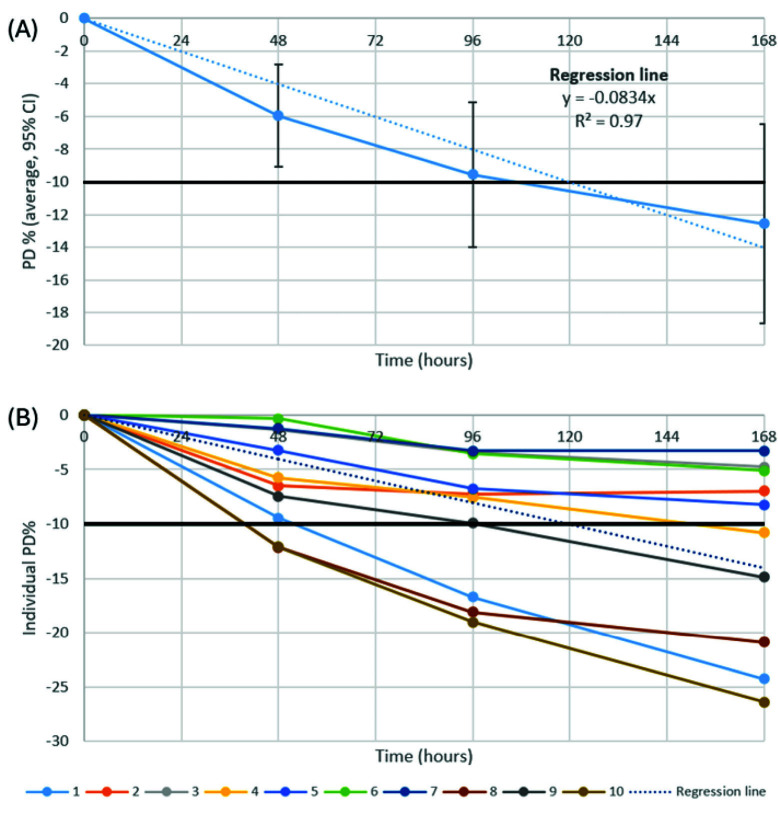
(A) Average PD% with 95% confidence intervals at each storage time point for PIGF stability measurement; (B) Individual PD% trajectories for each sample. The dashed line indicates the regression model and the solid black line marks the MPI threshold of - 10%. PIGF - placental growth factor. PD% - percent degradation. MPI - maximum permissible instability.

At 48 hours, mean degradation remained within the acceptable MPI threshold of - 10%, indicating adequate analyte stability. At 96 hours, however, the lower bound of the 95% CI crossed this threshold, suggesting that analyte stability may no longer be guaranteed. By 168 hours, a marked increase in degradation was observed and most samples exceeded the instability limit (Figure 3A).

Based on the linear regression equation, the estimated time at which PD% reaches the - 10% MPI threshold is approximately 120 hours (5 days) (Figure 3B).

**Figure 4:**
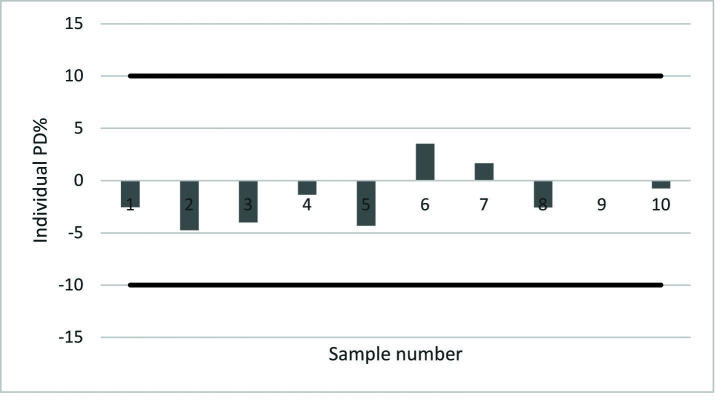
Individual PD% values after a double freeze-thaw cycle. PIGF - placental growth factor. PD% - percent degradation.

Finally, the effect of a double freeze-thaw cycle on PlGF stability was evaluated. The mean PD% following the second thaw was - 1.5%, with a 95% CI of - 3.4% to 0.4%, indicating minimal impact on stability (Figure 4).

## Discussion

This study provides the first structured assessment of urinary PlGF stability under conditions that simulate typical hospital workflows. Our results show that PlGF remains adequately stable at 2-8 °C for up to 48 hours, with the mean PD% and its 95% CI remaining within the predefined MPI of ± 10%. Beyond this time point, progressive degradation was observed, with the 95% CI at 96 hours exceeding the MPI threshold and most samples surpassing it at 168 hours. Additionally, PlGF remained stable after two freeze-thaw cycles, with a mean PD% of - 1.5% (95% CI: - 3.4% to 0.4%).

Stability studies on biomarkers have often used heterogeneous acceptance criteria, which limit comparability and reproducibility of results across laboratories (*15*, *17*, *18*). To improve consistency, the SEQC-ML proposed a structured protocol using MPI, PD% and regression modeling, which was followed in our study (*16*).

According to the consensus of the Milan Strategic Conference, clinical outcome-based specifications (model 1) could not be applied because no established clinical decision limits are available for urinary PlGF (*19*). Likewise, the biological variation-based approach (model 2) was not applicable due to the absence of urine-specific biological variation data. Although biological variation data exist for serum PlGF (intra-individual CV = 7.9%, inter-individual CV = 12.9%), suggesting an allowable bias of approximately 5.7%, these data were derived from non-pregnant women and PlGF concentrations are known to fluctuate with gestational age during pregnancy (*20*, *21*). Moreover, they refer to a different biological matrix, further limiting their relevance for the present study. Finally, the state-of-the-art approach (model 3) was also not applicable because no validated method is currently available for urinary PlGF measurement, as all existing immunoassays are designed for serum. Therefore, a pragmatic MPI threshold of ± 10% was adopted in the absence of previously reported urinary PlGF stability data and in line with previous thresholds used in serum PlGF stability studies (*13*, *14*, *22*, *23*).

The evidence available on PlGF stability is limited exclusively to studies performed in serum, which cannot be extrapolated to urine due to the fundamentally different biochemical composition of both matrices. Based on our results, urinary PlGF appears stable for at least 48 hours when stored at 2-8 °C. Beyond this period, degradation becomes more pronounced, with several samples exceeding the predefined instability limit, likely reflecting the intrinsic variability of urine, including differences in pH, ionic strength and the presence of proteases or bacterial enzymes. The degradation pattern observed is comparable to that of other urinary biomarkers, such as inflammatory proteins, which have also shown similar stability limitations at 4 °C (*9*, *24*). Overall, these findings highlight the need for further research on urinary PlGF stability, as no prior data exists and current knowledge from serum-based studies cannot inform urine-specific preanalytical behavior.

Recent studies have investigated urinary PlGF as a potential biomarker for PE, including its correlation with serum concentrations. Hebert-Schuster *et al.* reported a very strong correlation between urinary and serum PlGF concentrations (ρ = 0.983, P < 0.05), particularly when serum PlGF exceeded 100 pg/mL, suggesting that urinary PlGF may reliably reflect circulating concentrations (*25*). Similarly, Martín-Palumbo *et al.* found that urinary PlGF showed the strongest correlation with its serum counterpart (R^2^ = 0.73) among all markers studied and demonstrated high predictive value for PE (area under curve (AUC) = 0.866), comparable to that of serum PlGF (AUC = 0.853) (*26*). Although these studies support the clinical potential of urinary PlGF, none have addressed analyte stability, a key preanalytical requirement for reliable measurement. Demonstrating stability during refrigerated storage and over two freeze-thaw cycles is essential for its future implementation in both research and clinical workflows. The analyte’s acceptable performance under typical storage conditions supports its suitability for use in outpatient and non-specialized healthcare settings, where sample transport and delayed processing are common.

One of the main strengths of this study is its real-world applicability, as sample collection and handling were conducted under standard clinical laboratory conditions, reflecting typical preanalytical variability. Furthermore, applying a validated and structured stability protocol (SEQC-ML model) reinforces the methodological consistency and reliability of the results (*16*). All samples were processed in a single analytical batch, using the same reagent lot, minimizing analytical variability as much as possible. However, several limitations must be acknowledged. First, while the time-to-instability estimation (≈ 120 hours) derived from regression modeling provides useful insight, only the 48-hour time point showed consistent stability across all samples. Two of the ten aliquots exceeded the 10% MPI at 48 hours; however, according to the SEQC protocol, stability is determined by the mean PD% and its 95% CI rather than individual sample variability, reinforcing that the maximum acceptable stability is up to 48 hours. Second, there is currently no commercially available immunoassay and material controls for measuring urinary PlGF concentrations; available tests are validated only for serum. Lastly, although the sample size was sufficient for preliminary conclusions, a larger cohort would be necessary to confirm these findings.

In conclusion, urinary PLGF exhibits good stability under refrigerated conditions and shows minimal impact from freeze-thaw events, supporting its utility in clinical and translational research. Nevertheless, the sharp decline in stability beyond 48 hours and the high inter-sample variability emphasize the need for prompt processing and consistent preanalytical procedures. Our findings provide new preanalytical evidence on urinary PlGF stability, which is essential for its reliable implementation in clinical practice.

## Data Availability

The data generated and analyzed in the presented study are available from the corresponding author on request.
